# Using the Random Forest for Identifying Key Physicochemical Properties of Amino Acids to Discriminate Anticancer and Non-Anticancer Peptides

**DOI:** 10.3390/ijms241310854

**Published:** 2023-06-29

**Authors:** Yiting Deng, Shuhan Ma, Jiayu Li, Bowen Zheng, Zhibin Lv

**Affiliations:** 1 College of Biomedical Engineering, Sichuan University, Chengdu 610065, China; dengyt01@stu.scu.edu.cn (Y.D.); mashuhan820@foxmail.com (S.M.); zhengbowen101@foxmail.com (B.Z.); 2 College of Life Science, Sichuan University, Chengdu 610065, China; lijiayu927@foxmail.com

**Keywords:** random forest, anticancer peptide, amino acids index, physicochemical properties, feature selection

## Abstract

Anticancer peptides (ACPs) represent a promising new therapeutic approach in cancer treatment. They can target cancer cells without affecting healthy tissues or altering normal physiological functions. Machine learning algorithms have increasingly been utilized for predicting peptide sequences with potential ACP effects. This study analyzed four benchmark datasets based on a well-established random forest (RF) algorithm. The peptide sequences were converted into 566 physicochemical features extracted from the amino acid index (AAindex) library, which were then subjected to feature selection using four methods: light gradient-boosting machine (LGBM), analysis of variance (ANOVA), chi-squared test (Chi^2^), and mutual information (MI). Presenting and merging the identified features using Venn diagrams, 19 key amino acid physicochemical properties were identified that can be used to predict the likelihood of a peptide sequence functioning as an ACP. The results were quantified by performance evaluation metrics to determine the accuracy of predictions. This study aims to enhance the efficiency of designing peptide sequences for cancer treatment.

## 1. Introduction

Identifying novel anticancer compounds has long been a major focus of medical research. Conventional cancer treatments, such as surgery, radiotherapy, and chemotherapy, often adversely affect healthy cells and tissues and can lead to the development of treatment resistance. Therefore, it is essential to identify additional effective therapeutic options. Anticancer peptides (ACPs) are short peptide sequences that typically contain fewer than 50 amino acids. They exert their anticancer effects via a variety of mechanisms, for example, by inhibiting the proliferation and migration of cancer cells, inducing apoptosis, changing the pH in the internal and external cellular environment, or by damaging the cell membrane of cancer cells without affecting healthy tissues [1]. Compared to conventional therapeutic compounds, ACPs offer several advantages, such as high specificity, low intrinsic toxicity, high tissue permeability, and the convenience of sequence modification [2,3,4,5]. These features make ACPs a promising new therapeutic option in the management of cancer.

Historically, the identification of ACPs involved conventional laboratory experiments that were time-consuming and costly. However, with the accumulation of considerable ACP sequence data and the establishment of experimentally validated ACP databases, such as CancerPPD [2], DADP [6], CAMP [7], and APD [8], the rapid identification of novel ACP sequences using machine learning algorithms is becoming increasingly feasible. For example, in 2018, Wei et al. [9] used amino acid binary profile, amino acid type group, composition–transition–distribution, and twenty-one-bit features to represent peptides and then adopted a support vector machine learning method named ACPred_FL. In 2020, Rao et al. [10] developed another model named ACPred_Fuse, which applied 114 type features to represent peptides. In the same year, Agrawal et al. [11] used amino acid composition, dipeptide composition, terminus composition, and binary profile to develop an extra-tree-based model named AntiCP-2.0. With the rise in deep learning and protein representation learning, many new anticancer peptide recognition methods [11,12,13,14,15,16] (e.g., iACP-DRLF and TriNet) continue to emerge, and the performances of the methods are becoming increasingly better.

Nonetheless, current machine-learning-based ACP prediction models are limited. Analyzing standard datasets with alternative sequence feature extraction methods often results in greatly variable outcomes, and the causes of this divergence are currently unclear. In addition, studies to date have paid little attention to the key physicochemical features that differentiate ACPs from peptides with no ACP activity, resulting in an insufficient understanding of what features determine whether an amino acid sequence can act as an ACP. To overcome these shortcomings, we used the AAindex database [17,18,19,20] as the feature database of peptide sequences. This database consists of two subdatabases, AAindex1 and AAindex2. AAindex1 is a database representing the different physical, chemical, and biological properties of amino acids, currently containing 566 amino acid features. AAindex2 is an amino acid mutation matrix database that represents the similarity between amino acids, currently containing 94 matrices. The AAindex database is primarily used in protein-related research fields and as a machine learning database in protein prediction applications. However, the information contained within this database has not been previously utilized for extracting the key features of ACP sequences. Thus, no information is available on the physicochemical properties of key amino acids that affect the function of ACPs from a holistic perspective.

To address the problems of current ACP prediction models, we explored the physicochemical characteristics of amino acids predicting ACP activity. We combined multiple feature selection techniques and a random forest (RF) model to construct a single-feature model based on the physicochemical characteristics of ACP sequences [21,22,23]. The resulting model performed well in predicting ACPs in the calculation-based analysis of the physicochemical properties of key amino acids within the peptide sequences. This approach allowed us to identify 19 key amino acid properties that were useful in detecting ACP sequences in various benchmark datasets. A user-friendly webserver (https://www.aibiochem.net/servers/RFaaindexACP, accessed on 18 June 2023) is provided.

## 2. Results and Discussion

### 2.1. Analysis of Feature Selection Methods

Various feature selection methods can result in marked differences in the ranking of key features. Therefore, we compared the performance of the models using selected features with the baseline of non-selected 566-dimensional feature vector results.

#### 2.1.1. Model Performance Analysis before and after Feature Selection

**Figure 1:**
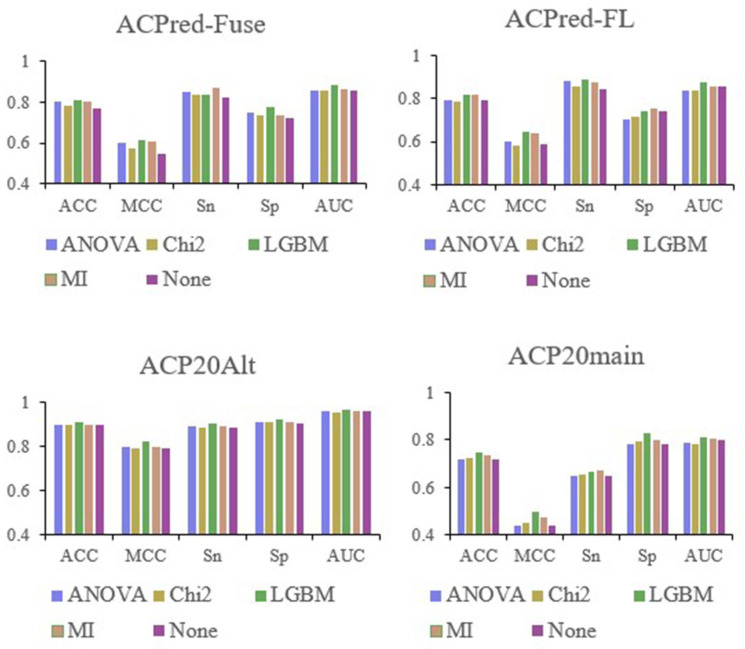
Comparison of performance metrics selected after the first feature selection.

After establishing an RF model based on 566-dimensional features, the four studied datasets, ACPred-Fuse, ACPred-FL, ACP20Alt, and ACP20main, were each processed using four different feature selection methods: ANOVA, Chi^2^, LGBM, and MI. Table 1 displays the optimized feature space dimensions for different datasets. For instance, for the ACPred_Fuse dataset, the best feature numbers were 73, 77, 28, and 40 after ANOVA, Chi^2^, LGBM, and MI were applied. A histogram was created for each index, as well as the baseline metrics without feature selection, and the corresponding ACC, MCC, Sn, Sp, and AUC values were compared, as shown in Figure 1. Irrespective of the dataset being analyzed, the best performing feature selection was invariably achieved using LGBM. Therefore, feature selection and optimization were carried out using this approach. 

#### 2.1.2. Feature Selection Results for Different Datasets Using the Same Feature Selection Method

After completing the feature selection steps using the four datasets, the key features obtained were compared longitudinally to identify overlaps. As shown in Table 1, the four distinct feature selection strategies resulted in a marked variation in the number of features being selected.

**Figure 2:**
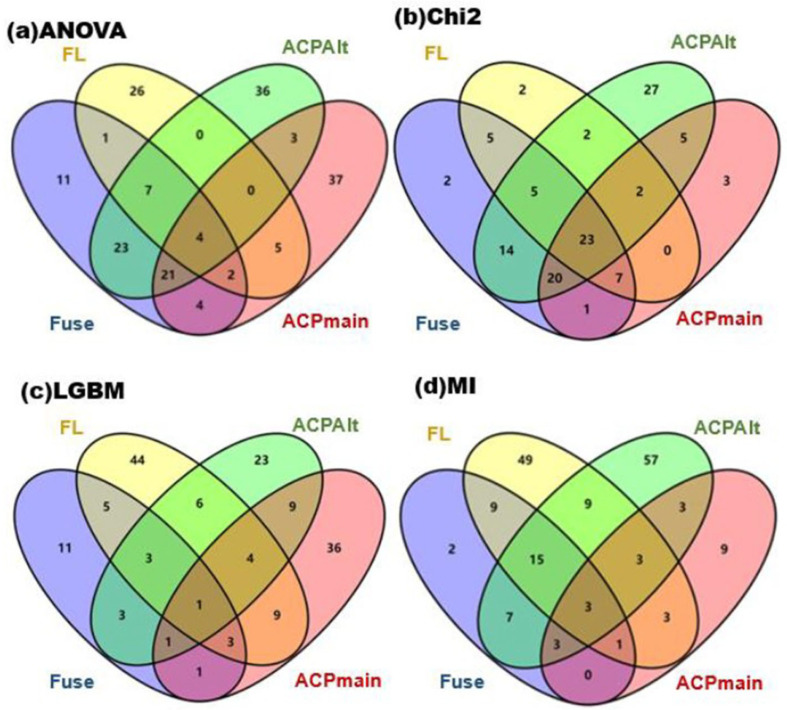
The intersection data derived from the four datasets.

Through data records, common features present in three or more datasets after each feature selection strategy were selected and analyzed using Venn diagrams, by intersecting the obtained features. These Venn diagrams, drawn using the Venny 2.1 online tool [24], are shown in Figure 2 (the tool is available at https://bioinfogp.cnb.csic.es/tools/venny/index.html, accessed on 1 January 2021).

In order to obtain universally applicable features and eliminate the influence of different feature selection methods, features obtained from the four datasets were intersected and merged. Since the number of features was relatively small, we only retained merged features common to at least three datasets. The number of common features obtained from the four feature selection methods were 27 features from ANOVA, 57 features from Chi^2^, 12 features from LGBM, and 25 features from MI. These were combined, obtaining a total of 105 features for the next round of feature selection.

The RF analysis results, using the 105 selected features, showed that the ACC, MCC, and AUC metrics for each dataset were almost optimal, with these metrics with three datasets being better than what could be achieved using the original, unselected data. However, when analyzing the ACP20Alt dataset, the performance of the selected features was slightly reduced, although the difference compared to the full 566-dimensional data was very small. Therefore, the selected 105 features appeared to be sufficient to identify ACPs in the analyzed datasets.

### 2.2. Model Analysis Based on 105 Selected Features

#### 2.2.1. Model Performance

In order to reduce the number of features further and make the selection results more representative, a second round of feature selection was conducted, starting with the 105 features described above. This round of feature selection was performed using LGBM on each of the four datasets, respectively. The results of this analysis are shown in Table 2. As shown in Table 2, the model based on the ACPred-Fuse dataset has the best independent test accuracy, while the model based on ACP20main has the smallest. Moreover, it can be concluded from Table 2 that the feature space dimensions of the models constructed on the basis of different datasets are different to obtain the best independent test accuracy. This means that these features are not sufficiently representative of all datasets.

#### 2.2.2. First Optimization Based on LGBM Feature Selection

**Figure 3:**
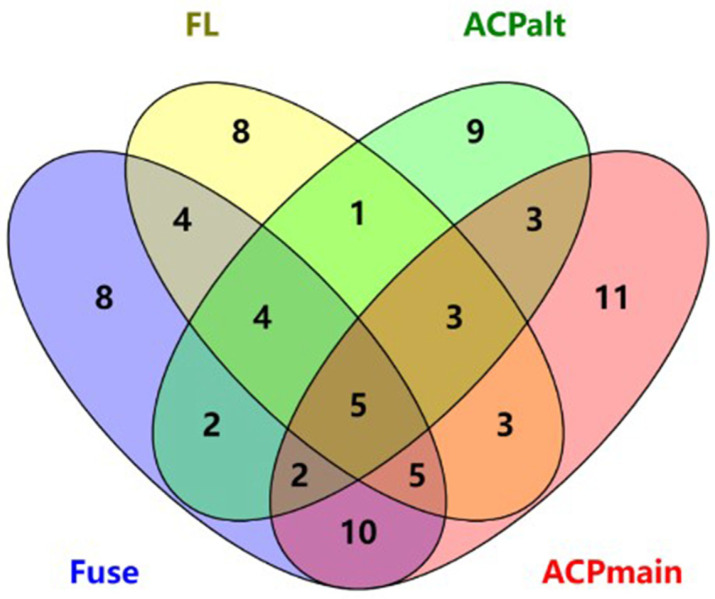
Intersection of features with a feature importance value greater than 0.01.

To obtain a common feature set represented by the four datasets, feature importance analysis was performed on the features obtained from the four datasets. Among the identified features, those with importance values greater than 0.01 were selected from the intersecting areas of the data. The intersection of the four dataset feature spaces is shown in a Venn diagram in Figure 3. Only 19 features satisfied these requirements.

#### 2.2.3. Second Optimization of the 19 Features by LGBM

**Figure 4:**
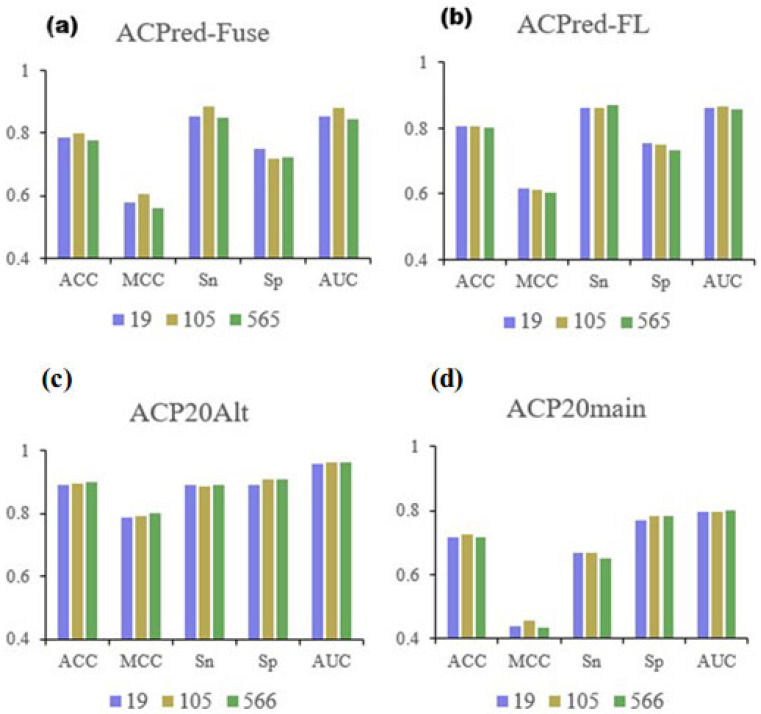
Comparison of performance metrics based on feature dimensionality. (**a**–**d**) is for metrics of models based on different datasets, respectively.

To explore how well the 19 selected features represented the full data, we created RF models using all four datasets. The cross-validation and independent testing results are shown in Table 3. We presented the results as histograms for the three feature selections and compared the three most important metrics: ACC, MCC, and AUC. The result of these comparisons is summarized in Figure 4. 

When the performance of the final 19 features was compared to the original 566-dimensional information or the previously selected 105 features, the results were very similar, indicating that the reduced feature set contained possibly all the necessary information to determine the characteristic properties of ACPs. During cross-validation, the performance metrics analyzing the ACPred-Fuse dataset showed some reduction, but the difference compared to the performance of the previously selected 105 features was small, indicating that the 19 features still captured sufficient information. In contrast, the metrics when testing the ACPred-FL dataset generally improved and were more representative. Comparing the performance of the full 566-dimensional data, the 105 and 19 selected features using peptide data from the ACP20Alt and ACP20main datasets only showed negligible differences, supporting the notion that the selected features were representative. The 19 physicochemical properties of amino acids from the AAindex database that were sufficient to predict ACP characteristics in peptides are shown in Table 4. 

Further, it shows pairwise correlations for all 19 physicochemical properties (see Supplementary Figures S7–S10). The diagonal lines of Figures S7–S10 show that there was a large overlap in the numerical distribution of all 19 features across the positive and negative samples. For a certain feature correlated to 18 other features, a two pairs graph showed that it was not enough to distinguish the anticancer and non-anti-cancer peptides well. These results meant that relying on the feature alone or a combination of two features is not enough to identify a peptide with anticancer activity from the peptide sequence, i.e., the primary structure. A fine numerical analysis of all 19 features must be relied upon to obtain better results. Figure S5 and the support material file show the random forest binary classification trees and forests. From Figure S5, it can be seen that through the fine division of each feature value, many binary classification judgments are formed, and finally, the peptides with anticancer activity can be concluded. Here, these 19 features shown in Table 4 detected most of the critical features in all four datasets; it is likely that they can be used to distinguish ACPs from peptides with no ACP activity in general. 

### 2.3. Statistical Comparison with Previously Reported Prediction Methods

We then compared the predictive performance of the RF-based prediction algorithm analyzing the initial model with 566-dimensional data versus those with 105- and 19- dimensional data and the previously reported ACP prediction methodologies. Obviously, the lack of statistically significant differences between the performance of the models would indicate that the 19 features captured sufficient information to distinguish ACPs from non-ACPs. The performance of the predictive algorithms previously reported in the literature is summarized in Table 5, while the comparison between the averaged performance of these previously reported approaches and our three models with 566-, 105-, and 19-dimensional features is shown in Table 6.

As shown in Table 6, the performance metrics of our predictive algorithm are generally lower than those for previously reported approaches. This is because of several reasons. The first is that the previously reported algorithms used two or more amino acid sequences in their feature representation; a multi-feature representation model will usually be better than a single-feature one. Here, in this study, we used a single feature (i.e., amino acid index), so the performance of the model will be slightly worse. Second, the focus of the previous algorithms was not the same as ours. The previous algorithms performed feature engineering optimization for a specific dataset. As a result, the optimized feature space of them usually only showed a better performance for that dataset, and the performance was worse for different datasets. That is, the generalization performance of the model was not good. Instead, we studied four standard datasets at the same time, looking for common feature representations that can be applied to different datasets, so as to build a more generalized model. Third, we also noticed a difference in performance compared to previous algorithms. For this reason, we performed a statistical significance test (see Table 7 and Table 8). The study showed that there was no statistically significant difference between the metrics of our results and the mean of the results optimized for specific datasets reported in the literature. This means that the 19 amino acid physicochemical properties we used can be applied to different datasets and obtain a performance without statistically significant difference from the literature algorithms.

## 3. Materials and Methods

**Figure 5:**
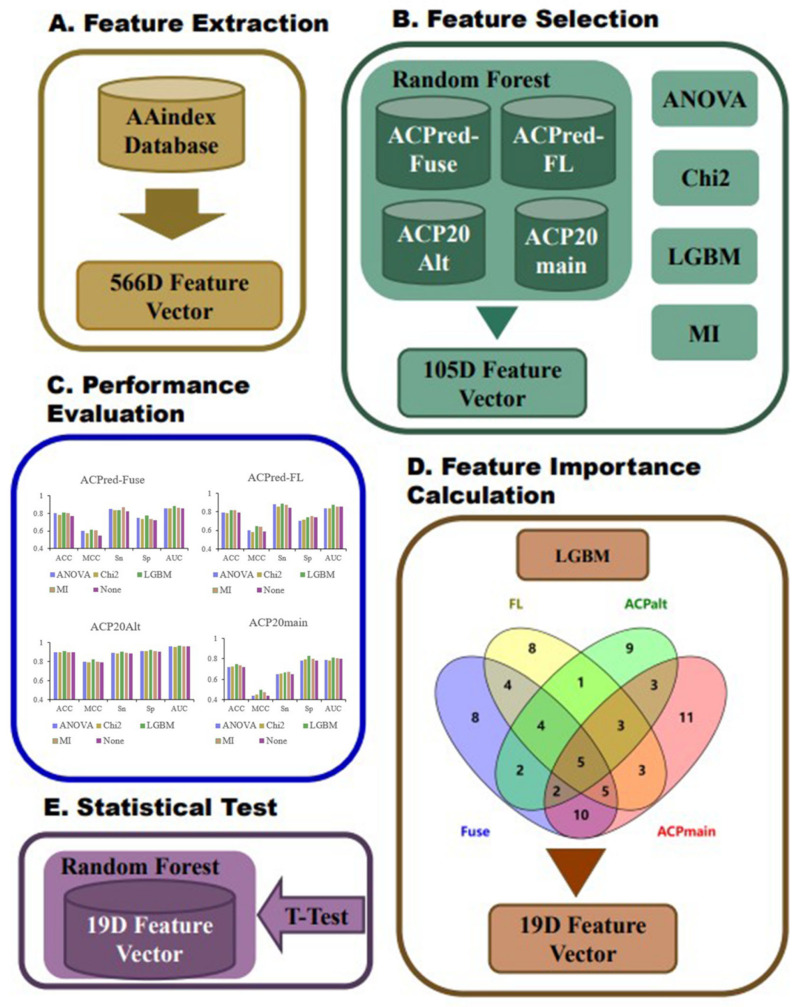
The overall flowchart of experimental design.

We constructed an amino acid sequence feature extraction tool based on the AAindex database, converting peptide amino acid sequences into 566-dimensional feature vectors, where each dimension represents a physicochemical property of an amino acid [25,26,27]. First, the ACP datasets were divided into training and test datasets, and we analyzed them by a random forest (RF) model based on the full 566-dimensional features of the AAindex database to select the most informative features. Next, we used light gradient-boosting machine (LGBM), analysis of variance (ANOVA), chi-squared test (Chi^2^), and mutual information (MI) analysis for feature selection. By examining the performance of the RF model based on the best top n features under the four methods, we initially selected 105 features. RF modeling was performed again based on these 105 features on the same benchmark datasets, and the best features were selected based on hyperparameter optimization and feature importance analysis. As a result, we identified the best performing top 19 features. Finally, the RF model was trained based on the 19 features using all four datasets. The best performing models were compared with previously reported ACP prediction algorithms described in the literature, and the statistical significance of the differences in prediction performance indices was calculated. The overall flowchart of the conducted work is summarized in Figure 5.

### 3.1. Benchmark Datasets

Throughout the work presented here, we used the ACP20Alt [28], ACP20main [28], ACPred-FL [9], and ACPred-Fuse [10] datasets. Of these, the positive samples of ACPred-FL and ACPred-Fuse were primarily published by Chen et al. [29], and Tyagi et al. [2] in their CancerPPD datasets. Peptides with no ACP activity were represented by antimicrobial peptides (AMPs) and by a collection of peptides that had no anticancer effect during experimental testing. We randomly selected 250 ACP and 250 non-ACP sequences from the ACPred-FL dataset to act as the training dataset, and 82 ACPs and 92 non-ACPs as the test dataset. The 250 positive samples in the ACPred-Fuse training dataset were selected from the work of Wei et al. [9] Half of the 250 negative samples were also derived from Wei et al., while the other half was collected from the AMP dataset. The test dataset contained all the remaining ACPred-Fuse data (82 ACPs and 2628 non-ACPs) as positive and negative samples. The ACP20main and ACP20Alt databases were compiled by Lv et al. [28]. ACP20main contains 861 experimentally verified ACPs and 861 peptides with no documented ACP activity. Peptides from this dataset were divided into two subsets for 5-fold cross-validation and independent testing. Finally, the ACP20Alt database contains 970 ACPs and 970 non-ACPs. These were also subdivided into a training set and an independent test subset. The main difference between the ACP20main and ACP20Alt databases is that the negative samples in the former are represented by AMPs, while the negative samples in ACP20Alt are randomly selected peptides, assumed to have no antitumor activity. The main details of the used benchmark datasets are summarized in Table 9. The shared sequences numbers are shown in Figure S6 in the supporting information.

### 3.2. Feature Extraction

To investigate the physicochemical properties of key amino acids, we first had to extract the information contained in the ACP sequences and convert raw sequences into interpretable features [30,31]. The data for this feature extraction were obtained from the AAindex databases. The AAindex1 database, available at https://www.genome.jp/aaindex/ [32] (accessed on 1 January 2020), describes the physicochemical properties of amino acids using 566 indices. 

The physicochemical properties of a peptide sequence with a length of *L* were extracted from the amino acid index database as numerical values for each amino acid [33,34]. It means that each amino acid in the peptide sequence is represented by a 566-dimensional vector, and then a peptide sequence of *L* amino acids will be transformed into an *L* × 566 matrix. Next, an average pooling of the matrix is conducted, resulting in a 566-dimensional vector representing the peptide sequence. This approach transformed each peptide sequence into a 566-dimensional feature vector, where each dimension represents a particular physicochemical property of amino acids [21]. The source code for peptides from the AAindex feature extraction can be downloaded from https://github.com/zhibinlv/RFaaindexACP (accessed on 19 June 2023).

### 3.3. Feature Selection Methods

Feature selection is the process of filtering the most relevant features by machine learning [35]. The main purpose of feature selection is to identify irrelevant or redundant features, reducing the runtime of machine learning algorithms and obtaining more accurate results. Four feature selection methods were used in our research: light gradient-boosting machine (LGBM), analysis of variance (ANOVA), chi-squared test (Chi^2^), and mutual information (MI). The LGBM feature selection code can be found at https://github.com/zhibinlv/iACP-DRLF/tree/main/feature_selection (accessed on 1 January 2020). For Chi^2^, ANOVA, and MI, it can be found using the scikit-learn toolkits at https://scikit-learn.org/stable/index.html (accessed on 1 January 2020).

#### 3.3.1. LGBM

LGBM adopts the histogram algorithm [36], in which continuous features are turned into *k* discrete values in order to construct a histogram with a width of *k*. The algorithm is trained to count the value of each discrete value in the histogram. Based on these discrete values, the optimal feature segmentation points can be determined, and the exact number of key features can be obtained.

#### 3.3.2. ANOVA

ANOVA analyzes the relationship between independent variables and dependent variables by studying whether the variance of multiple samples is equal to the overall mean value [37,38]. It can perform feature extraction before the data enter classifier training, thus reducing data dimensionality. Taking binary classification as an example, using ANOVA for feature selection divides the value of a certain feature into two groups, a positive and a negative group. The greater the difference between these groups in ANOVA, the greater their impact on the sample. 

#### 3.3.3. Chi-Squared Test 

A chi-squared test can be used to determine whether two variables are correlated and to calculate the extent of this correlation [39], by performing a test between the feature and the real label. Assuming that the number of independent variables is *A*, and the number of dependent variables is *B*, the following equation *χ* can be constructed:

(1) $$\;\chi^{2}=\sum \frac{{\left(A-B\right)}^{2}}{B}\;$$

The result indicates the degree of dependence between the independent variables and dependent variables. 

#### 3.3.4. MI

Mutual information [40,41] can also be used to test the correlation between two variables, which can be defined as follows:

(2) $$I(X,\;Y)=\underset{y\in Y}{\sum }\;\underset{x\in X}{\sum }\;p(x,\;y)\log(\frac{p(x,\;y)}{p(x)p(y)})$$

If *x* and *y* are random variables that are independent of each other, then

(3) $$\;p\left(x,y\right)=p\left(x\right)p\left(y\right)\;$$

Thus, the result of *I*(*X*, *Y*) is 0. Therefore, a larger *I*(*X*, *Y*) indicates a greater correlation between the two variables, allowing feature filtering.

### 3.4. Random Forest Algorithm

In this study, we used a random forest (RF) [42,43,44,45] machine learning algorithm to analyze the importance of features. By determining the contribution of each feature to each tree in the models, the importance of key features can be ranked, identifying key amino acid physicochemical properties that determine the likelihood of a given peptide having ACP activity. 

During this process, the RF model selects several samples from the sample set to construct a training dataset with replacement. It then uses the obtained training dataset to generate a decision tree and randomly selects multiple non-repetitive features at each node, using these features to divide the sample set [46]. After the optimal division features are found, the process is repeated until all the decision trees in a random forest are generated [47]. Finally, the model trained by the above steps is used to predict the sample set, and the prediction result is determined by the number of classifications. 

### 3.5. Feature Importance Analysis

Common evaluation metrics for calculating feature importance include the *Gini* index [48] and the out-of-bag (OOB) error. The *Gini* index is calculated according to the following equation:

(4) $$Gini\left(p\right)=\overset{k}{\underset{k=1}{\sum }}p_{k}\left(1-p_{k}\right)=1-\overset{k}{\underset{k=1}{\sum }}p_{k}^{2}$$

where k indicates the number of features divided into k categories, and $p_{k}$ represents the importance of category k.

The OOB dataset refers to the data that are not chosen in the sampling process. For a tree in the RF model, the error $e_{1}$ is obtained by the out-of-bag data sample, and then, error $e_{2}$ is derived by randomly permuting the *i*-th column of the out-of-bag data matrix. This way, the importance of feature *i* can be represented by $e_{1}-e_{2}$:

(5) $$Imp_{i}=\frac{\sum \left(e_{1}-e_{2}\right)}{n}\;$$

where *n* represents the number of decision trees in the model.

### 3.6. Evaluation of Model Performance

To evaluate the performance of each model, the sensitivity (*Sn*), specificity (*Sp*), accuracy (*ACC*), Matthews correlation coefficient (*MCC*), and area under receiver-operating characteristic curve (AUC) [49,50] were calculated as follows:

(6) $$\;Sn=\frac{TP}{TP+FN}\times 100\%\;$$

(7) $$\;Sp=\frac{TN}{TN+FP}\times 100\%\;$$

(8) $$\;ACC=\frac{TP+TN}{TP+FP+FN+TN}\times 100\%$$

(9) $$\;MCC=\frac{TP\times TN-FP\times FN}{\sqrt{\left(TP+FP\right)\times \left(TP+FN\right)\times \left(TN+FP\right)\times \left(TN+FN\right)}}\;$$

*TP*, *TN*, *FP*, and *FN* represent the number of true positive, true negative, false positive, and false negative samples, respectively. In addition, we also analyzed the ROC curve and AUC area, where a larger AUC value indicates a stronger predictive performance.

### 3.7. Websever

After the model was developed and optimized, we developed a simple and easy-to-use website for interested readers to use. The reader only needs to enter the peptide sequence in FASTA format and after a few minutes can find out whether those peptides have anticancer activity. The website can be found at https://www.aibiochem.net/servers/RFaaindexACP (accessed on 18 June 2023). A simple screenshot of the application is shown in Figures S1–S4.

## 4. Conclusions

The work presented in this paper proposed to identify the key physicochemical properties of ACPs based on existing machine learning algorithms, using existing ACP datasets available in the literature and amino acid features collated in the AAindex database. We analyzed the influence of four feature selection methods, ANOVA, Chi^2^, LGBM, and MI, on identifying key features. The comparison of these methods revealed that LGBM was the best approach for selecting features that led to the creation of the best fitting RF model and resulted in the best performance indices. Ultimately, this work identified 19 key amino acid features, which compared favorably with machine learning models reported previously in the literature. Furthermore, statistical tests revealed that the 19 key identified features provided as much information as predictions based on a much larger feature dimensionality or alternative machine learning algorithms, enhancing the credibility of our approach. Based on these 19 key features, we can develop new machine learning models with better effects or further refine existing models. In addition, based on these key properties, investigators will be able to design prospective ACPs with an improved probability of therapeutic effectiveness, thereby increasing the speed of transitioning peptides into clinical ACP research.

## Figures and Tables

**Table 1:** The number of features selected from the four datasets using the indicated feature selection methods.

Datasets	ANOVA	Chi^2^	LGBM	MI
ACPred-Fuse	73	77	28	40
ACPred-FL	45	46	75	92
ACP20Alt	94	98	50	100
ACP20main	76	61	64	25

**Table 2:** The metrics of selected features used to create the 105-feature matrix.

Datasets	Number of Features	5-Fold Cross-Validation					Independent Test				
		ACC (/%)	MCC	Sn (/%)	Sp (/%)	AUC	ACC (/%)	MCC	Sn (/%)	Sp (/%)	AUC
ACPred-Fuse	58	80.2	0.612	88.4	72.0	0.858	92.9	0.457	93.9	70.7	0.897
ACPred-FL	33	80.0	0.601	83.2	76.8	0.863	76.2	0.528	81.7	70.7	0.856
ACP20Alt	61	89.7	0.794	89.2	90.2	0.960	90.9	0.819	89.1	92.7	0.960
ACP20main	80	74.2	0.489	67.2	81.2	0.800	69.3	0.386	67.3	71.3	0.792

**Table 3:** Performance metrics using the final 19 selected features.

Datasets	Number of Features	5-Fold Cross-Validation					Independent Test				
		ACC (/%)	MCC	Sn (/%)	Sp (/%)	AUC	ACC (/%)	MCC	Sn (/%)	Sp (/%)	AUC
ACPPred-Fuse	19	78.6	0.576	84.8	72.4	0.854	93.0	0.447	94.1	68.3	0.900
ACPred-FL	19	80.6	0.616	86.0	75.2	0.860	79.9	0.601	85.4	74.4	0.846
ACP20Alt	19	89.3	0.786	89.3	89.3	0.958	89.4	0.791	85.0	93.8	0.952
ACP20main	19	71.9	0.440	66.7	77.0	0.797	70.5	0.410	68.4	72.5	0.789

**Table 4:** List of the selected 19 key features and the corresponding physicochemical properties.

Feature	Physicochemical Properties
QIAN880113	Weights for alpha-helix at the window position of 6
QIAN880126	Weights for beta-sheet at the window position of 6
BROC820102	Retention coefficient in HFBA
RACS820101	Average relative fractional occurrence in A0(i)
SNEP660103	Principal component III
OOBM850104	Optimized average non-bonded energy per atom
SNEP660104	Principal component IV
FINA910103	Helix termination parameter at position j-2, j-1,j
OOBM850101	Optimized beta-structure-coil equilibrium constant
RICJ880110	Relative preference value at C5
RICJ880112	Relative preference value at C3
CHAM830102	A parameter defined from the residuals obtained from the best correlation of the Chou–Fasman parameter of beta-sheet
ZASB820101	Dependence of partition coefficient on ionic strength
KLEP840101	Net charge
FINA910101	Helix initiation parameter at position i-1
MEEJ800101	Retention coefficient in HPLC, pH7.4
WOLS870103	Principal property value z3
AURR980112	Normalized positional residue frequency at helix termini C4
KARP850103	Flexibility parameter for two rigid neighbors

**Table 5:** Comparison of previously reported machine learning algorithms from the literature.

	5-Fold Cross-Validation					Independent Test				
	ACC (/%)	MCC	Sn (/%)	Sp (/%)	AUC	ACC (/%)	MCC	Sn (/%)	Sp (/%)	AUC
ACPPred-Fuse	82.4	0.652	77.2	87.6	0.882	89.0	0.320	72.0	89.5	0.868
ACPred-FL	89.0	0.783	84.8	93.2	0.940	87.8	0.758	84.1	91.5	0.960
ACP20Alt	93.0	0.861	89.7	96.3	0.978	92.5	0.852	88.6	96.4	-
ACP20main	76.7	0.536	72.2	81.1	0.841	73.4	0.468	76.0	70.8	-

**Table 6:** Comparison of the averaged literature results with the performance of RF based on three feature selections.

	5-Fold Cross-Validation					Independent Test				
	ACC (/%)	MCC	Sn (/%)	Sp (/%)	AUC	ACC (/%)	MCC	Sn (/%)	Sp (/%)	AUC
566D	82.6	0.654	85.8	79.3	0.891	85.8	0.578	86.5	78.9	0.904
105D	80.7	0.616	81.6	79.7	0.876	82.1	0.541	81.7	78.0	0.874
19D	80.1	0.604	81.7	78.5	0.867	83.2	0.562	83.2	77.2	0.872
Literature average	85.3	0.708	81.0	89.6	0.910	85.7	0.5995	80.2	87.1	-

**Table 7:** Results of statistical tests based on 5-fold cross-validation with a significance level α = 0.05.

Comparison of Results	ACC	MCC	Sn	Sp	AUC
105D vs. 566D	0.538	0.538	0.466	0.612	0.606
19D vs. 566D	0.435	0.437	0.473	0.339	0.375
19D vs. 105D	0.182	0.169	0.795	0.129	0.229
566D vs. Literature results	<0.001	0.449	<0.001	<0.001	0.548
105D vs. Literature results	<0.001	0.043		<0.001	0.115
19D vs. Literature results	<0.001	0.018	<0.001	<0.001	0.047

**Table 8:** Results of statistical tests based on an independent test with a significance level α = 0.05.

Comparison of Results	ACC	MCC	Sn	Sp	AUC
105D vs. 566D	0.417	0.464	0.4	0.602	-
19D vs. 566D	0.589	0.786	0.572	0.433	-
19D vs. 105D	0.433	0.35	0.416	0.528	-
566D vs. Literature results	0.983	0.792	0.317	0.298	-
105D vs. Literature results	0.279	0.424	0.818	0.186	-
19D vs. Literature results	0.383	0.575	0.677	0.175	-

**Table 9:** The number of ACPs and non-ACPs in the four datasets derived from the literature.

Datasets	Training Dataset		Testing Dataset		Sequence Similarity	Sequence Length (L)	Average
	ACP	Non-ACP	ACP	Non-ACP			Length
ACPred-Fuse	125	125	82	1871	<80%	7 ≤ L ≤ 207	25
ACPred-FL	250	250	82	82	<90%	11 ≤ L ≤ 207	27
ACP20Alt	775	775	193	193	<80%	2 ≤ L ≤ 50	24
ACP20main	688	688	171	171	<80%	3 ≤ L ≤ 50	24

## Data Availability

The data used to support the findings of this study can be made available by the corresponding author upon request.

## Supplementary Materials

The following supporting information can be downloaded at https://www.mdpi.com/article/10.3390/ijms241310854/s1.
