# Supplementary material for: Using the Random Forest for Identifying Key Physicochemical Properties of Amino Acids to Discriminate Anticancer and Non-Anticancer Peptides
Source: Int J Mol Sci. 2023 Jun 29;24(13):10854. doi: 10.3390/ijms241310854 (PMC10341712; doi:10.3390/ijms241310854)
Supplement: Supplementary file 1 [file ijms-24-10854-s001.zip › ijms-2414352-supplementary.pdf]

Supporting Information

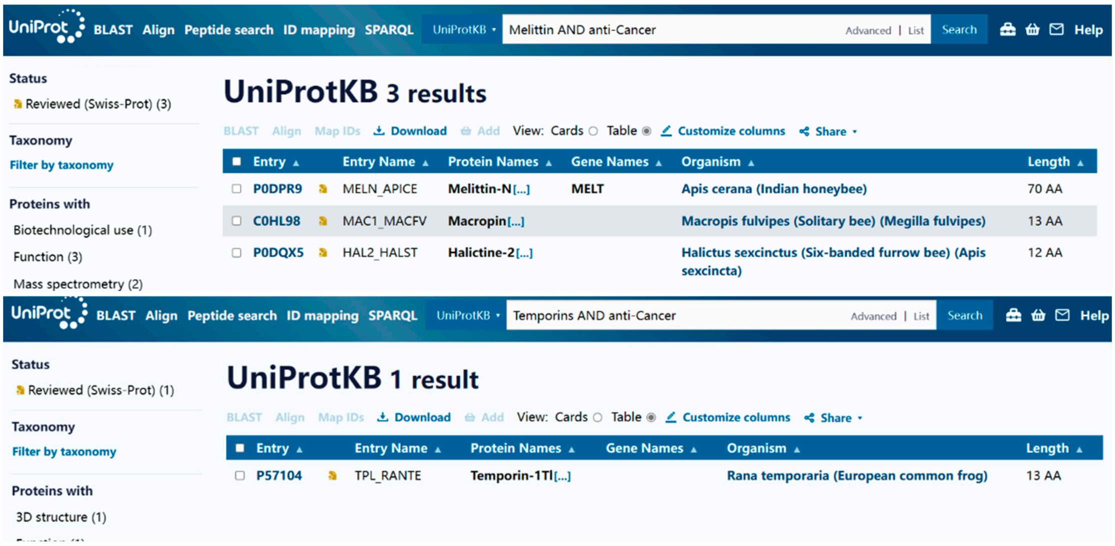

Figure S1. Search Melittin and Temporins in UniProt

```
>sp|C0HL98|MAC1_MACFV Macropin OS=Macropis fulvipes OX=465486 PE=1 SV=1 Melittin
GFGMALKLLKKVL
>sp|P0DQX5|HAL2_HALST Halictine-2 OS=Halictus sexcinctus OX=115105 PE=1 SV=1 Melittin
GKWMSLLKHILK
>sp|P0DPR9|MELN_APICE Melittin-N OS=Apis cerana OX=7461 GN=MELT PE=1 SV=1 Melittin
MKFLVNVALVFMVVYISYIYAAPEPEPAPEPEAEADAEADPEAGIGAVLKVLTTGLPALINWIKRKRQQG
>Melittin|HY-P0233
GIGAVLKVLTTGLPALISWIKRKRQQ
>sp|P57104|TPL_RANTE Temporin-1Tl OS=Rana temporaria OX=8407 PE=1 SV=1|
FVQWFSKFLGRIL
```

Figure S2. Five sequences data of Melittin and Temporins from UniProt and https://www.medchemexpress.cn/**. They are stored in FASTA format.**

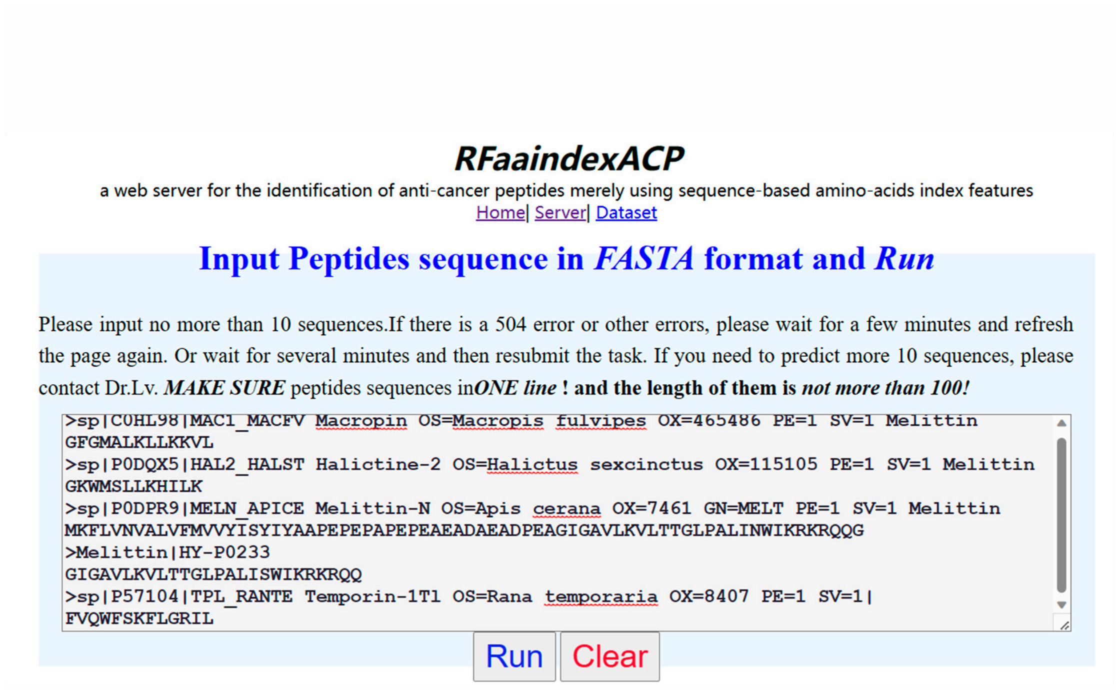

Figure S3. Five sequences data of Melittin and Temporins input in the webserver.

RFaaindexACP

a web server for the identification of ANTI-CANCER peptides merely using AAindex features

Home| Server| Dataset

Results

SEQUENCES	ANTI-CANCER PEPTIDES	CONFIDENCE
GFGMALKLLKKVL	YES	65.81%
GKWMSLLKHILK	YES	67.57%
MKFLVNVALVFMVVYISYIYAAPEPEPAPEPEAEADAEADPEAGIG...	YES	52.92%
GIGAVLKVLTTGLPALISWIKRKRQQ	YES	60.21%
FVQWFSKFLGRIL	YES	65.57%

Figure S4. The predict results of five sequences data of Melittin and Temporins are shown in the webserver.

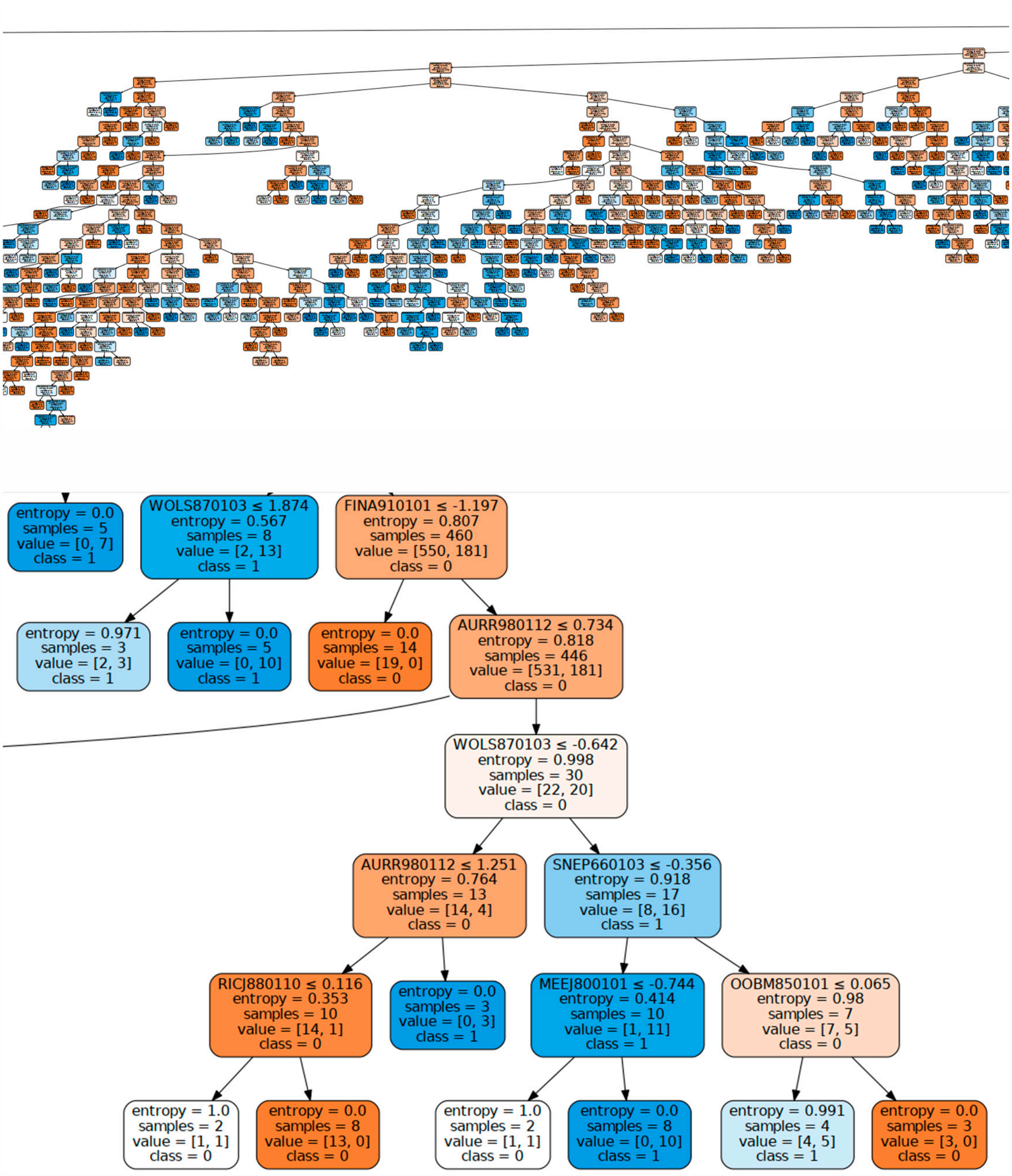

Figure S5. Local magnification of binary tree in random forest. The global view of the random forest was shown in the supporting materials named GolbalViewOfRF.pdf.

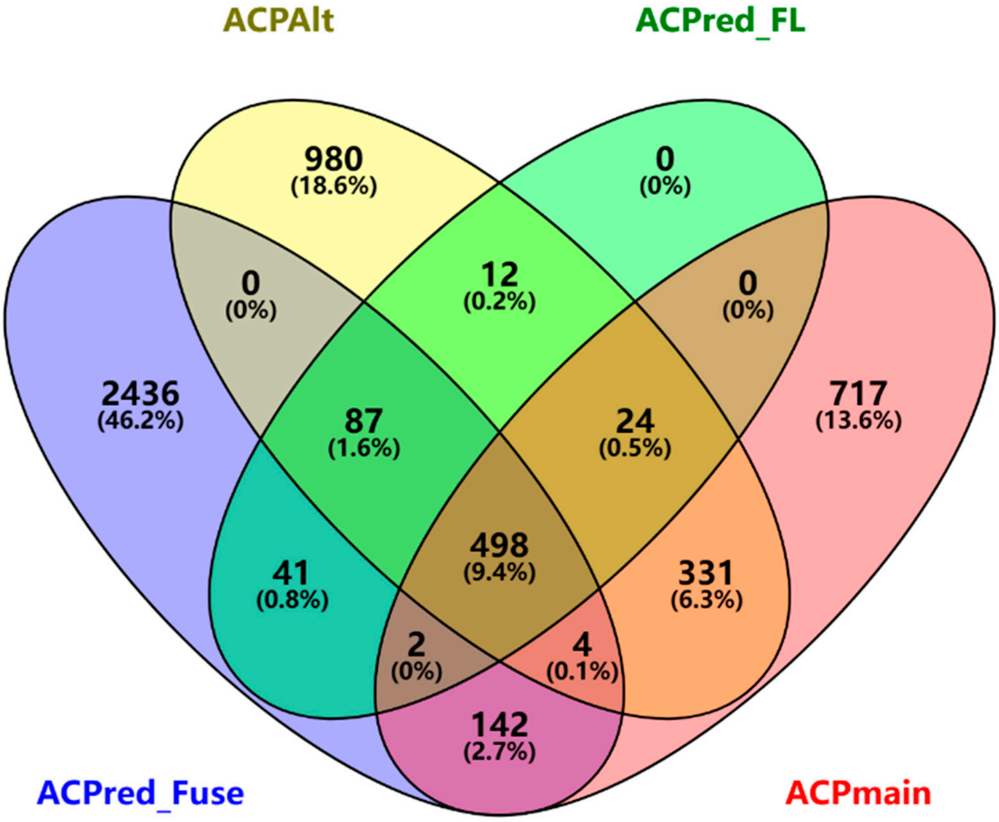

Figure S6. Venn diagram of four benchmark data used in this study. Intersecting sections represent peptide sequences that are common between datasets.

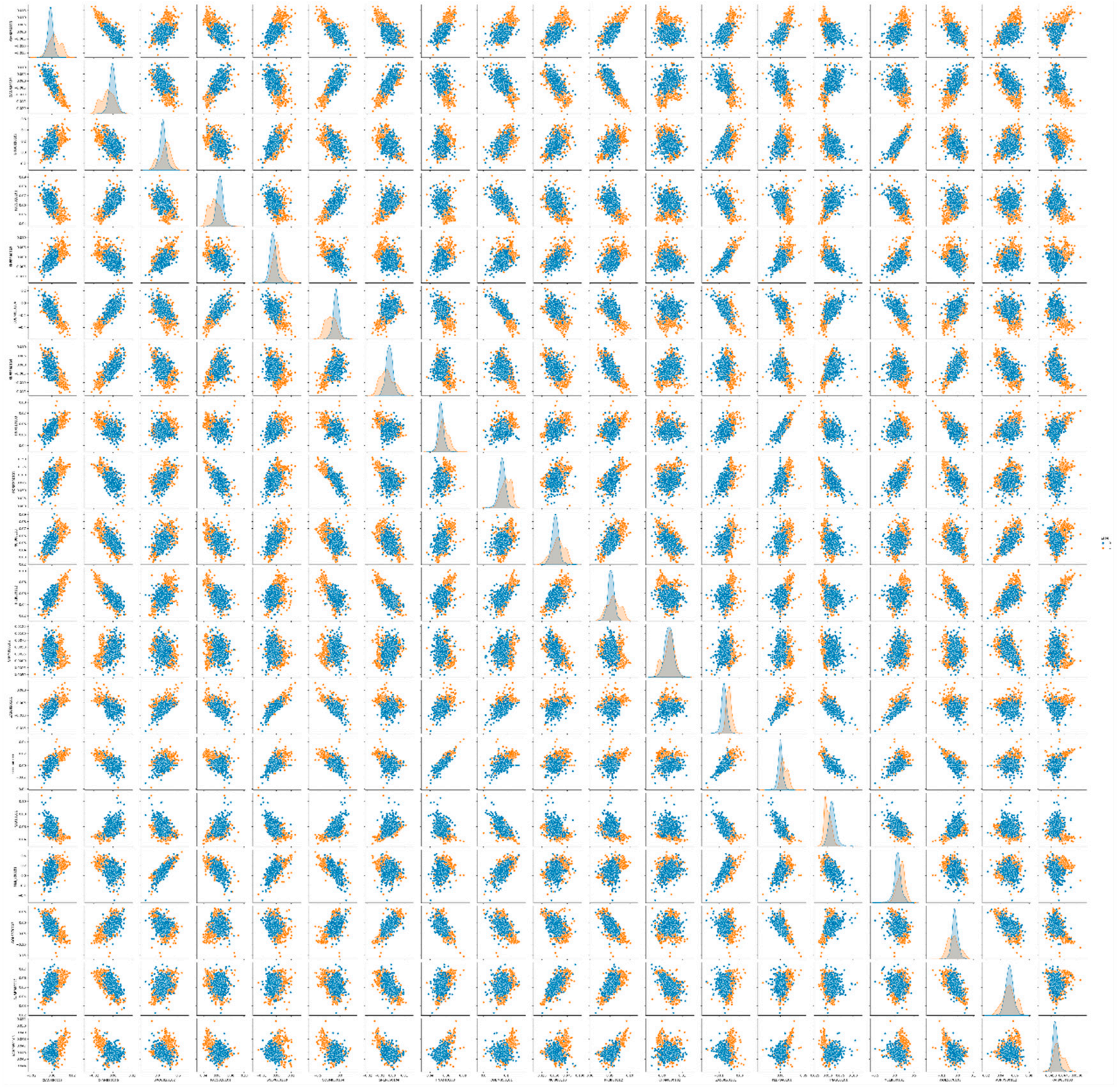

Figure S7. For the feature data of ACPalt dataset, 19 feature pairwise relationship joint distribution maps.

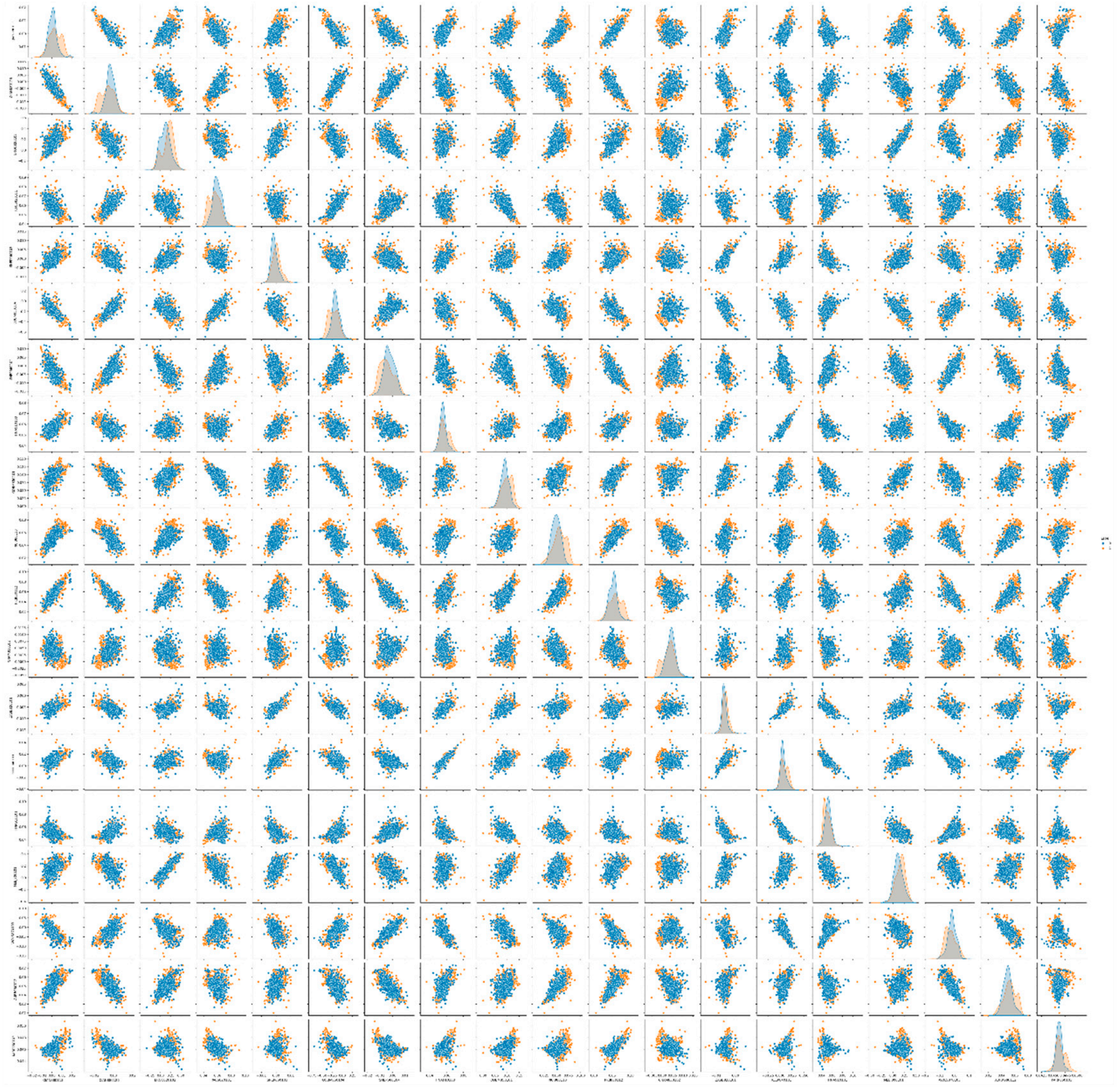

Figure S8. For the feature data of ACPmain dataset, 19 feature pairwise relationship joint distribution maps

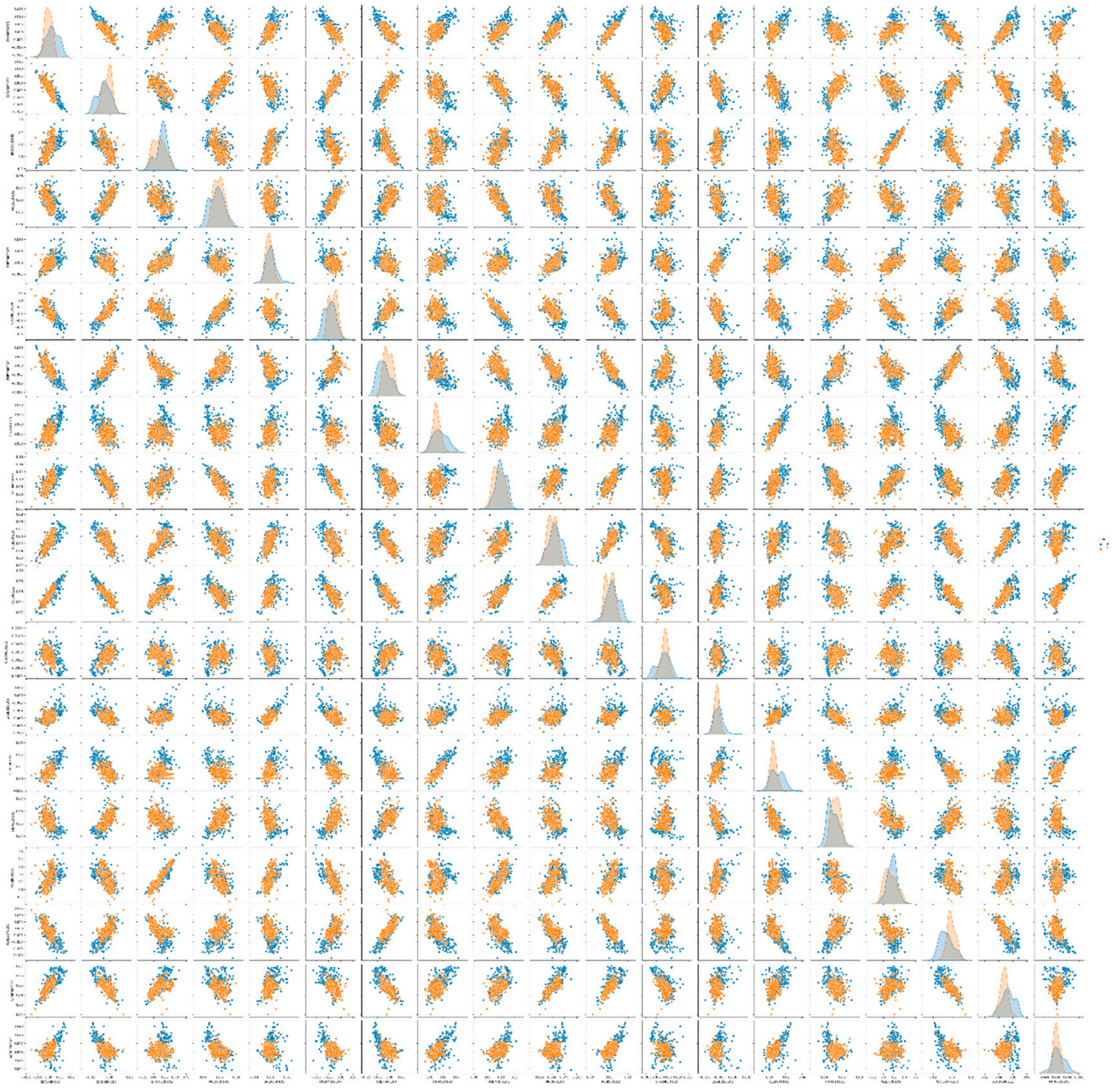

Figure S9. For the feature data of ACPred_FL dataset, 19 feature pairwise relationship joint distribution maps.

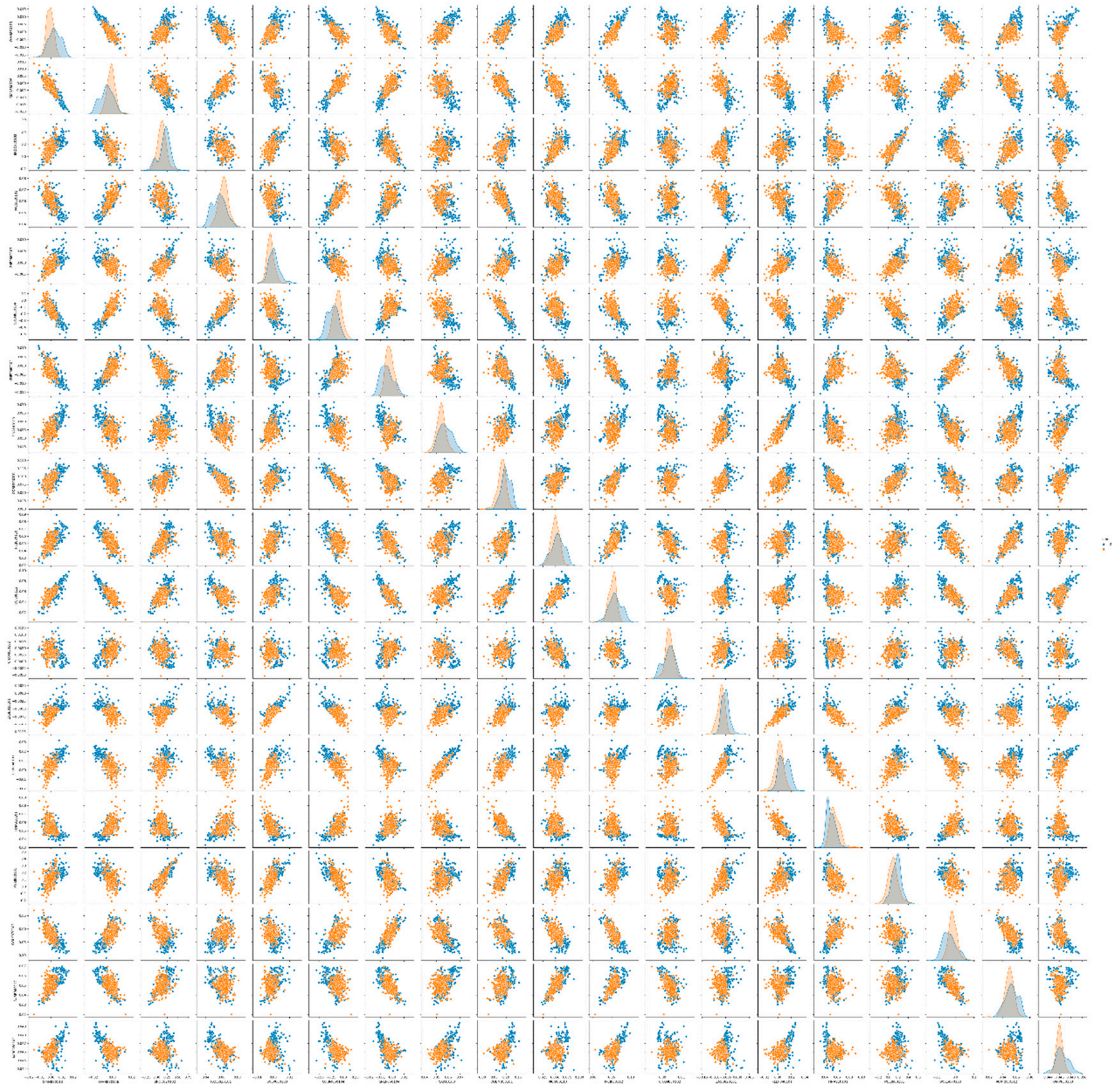

Figure S10. For the feature data of ACPred_Fuse dataset, 19 feature pairwise relationship joint distribution maps.
